# The Ubiquitin Ligase CBLC Maintains the Network Organization of the Golgi Apparatus

**DOI:** 10.1371/journal.pone.0138789

**Published:** 2015-09-22

**Authors:** Wan Yin Lee, Germaine Goh, Joanne Chia, Adrian Boey, Natalia V. Gunko, Frederic Bard

**Affiliations:** 1 Institute of Molecular and Cell Biology, Singapore, Singapore; 2 Institute of Medical Biology, Singapore, Singapore; 3 Department of Biochemistry, National University of Singapore, Singapore, Singapore; 4 IMB-IMCB Joint Electron Microscopy Suite, Singapore, Singapore; Institut Jacque Monod, Centre National de la Recherche Scientifique, FRANCE

## Abstract

The Golgi apparatus plays a pivotal role in the sorting and post-translational modifications of secreted and membrane proteins. In mammalian cells, the Golgi is organized in stacks of cisternae linked together to form a network with a ribbon shape. Regulation of Golgi ribbon formation is poorly understood. Here we find in an image-based RNAi screen that depletion of the ubiquitin-ligase CBLC induces Golgi fragmentation. Depletions of the close homologues CBL and CBLB do not induce any visible defects. In CBLC-depleted cells, Golgi stacks appear relatively unperturbed at both the light and electron microscopy levels, suggesting that CBLC controls mostly network organization. CBLC partially localizes on Golgi membranes and this localization is enhanced after activation of the SRC kinase. Inhibition of SRC reverts CBLC depletion effects, suggesting interplay between the two. CBLC’s regulation of Golgi network requires its ubiquitin ligase activity. However, SRC levels are not significantly affected by CBLC, and CBLC knockdown does not phenocopy SRC activation, suggesting that CBLC’s action at the Golgi is not direct downregulation of SRC. Altogether, our results demonstrate a role of CBLC in regulating Golgi ribbon by antagonizing the SRC tyrosine kinase.

## Introduction

The mammalian Golgi apparatus displays a unique and striking ribbon-like structure that has long fascinated cell biologists. Golgi organization is thought to be required for at least some of its many functions, such as post-translational modifications, including protein glycosylation, of membrane and secreted proteins and lipids, and cargo sorting to various cellular destinations.

The basic Golgi units are flattened, disk-shaped membrane-bound compartments known as cisternae, organized in stacks. In a typical mammalian Golgi, four to eleven cisternae are piled up to form a polarized cis-to-trans stack, with cisternal content and structure varying from one side to the other [1, 2]. The stacks are in turn interconnected on their sides through tubular structures to form a continuous, ribbon-like network localized in a perinuclear region [3].

The Golgi ribbon does not exist in lower eukaryotes such as the budding yeast *S*. *cerevisiae* or in simpler metazoan such as *D*. *melanogaster* [4, 5]. A Golgi network is only found in vertebrates, with the stacks of plant and insect cells being distributed throughout the cytoplasm. Perhaps not surprisingly then, the ribbon structure is not necessary for basic membrane trafficking and its disruptions minimally affect intra-Golgi trafficking and general secretion to the plasma membrane [6–9]. However, networking of Golgi stacks could be required for protein glycosylation and polarized cell migration [8, 10].

Despite the organizational complexity of this organelle, it is increasingly appreciated that the Golgi is quite plastic and dynamic. For example, its morphology changes during cell mitosis [11], cell migration, in which the whole organelle orients towards the direction of cell movement [10], and apoptosis, during which it fragments into dispersed stacks [12]. Given its role in many dynamic cell processes, it is not surprising that the Golgi is subject to considerable regulation in response to environmental as well as cell-intrinsic cues. Indeed, recent work has uncovered a number of regulatory mechanisms controlling the organization and function of the Golgi [13–15]. For example, ERK signaling controls the reorientation of the Golgi towards the leading edge during cell migration [16] and heterotrimeric GTPases and SRC signaling have been proposed to allow the Golgi to adjust to changes in secretory cargo load [9, 17].

In addition, it has also emerged recently that protein glycosylation can be regulated via modulation of Golgi organization. A systematic RNAi study suggests that multiple signaling proteins can regulate glycosylation pathways through modulation of Golgi organization [15]. SRC signaling has also been implicated in this process: growth factor activation of SRC triggers a retrograde movement of O-glycosylation initiation enzymes from the Golgi to the ER and a consequent increase in O-glycosylated proteins [18]. This process is regulated by various other signaling proteins and promotes cell adhesion and tissue invasion [19, 20].

In this study, we report that a pilot RNAi screen of Golgi organization revealed that depletion of the ubiquitin ligase CBLC caused extensive Golgi fragmentation. Cbl proteins have been established as multivalent adaptor proteins as well as E3 ubiquitin ligases. For instance, the better known isoform CBL has been shown to interact with over 150 proteins via its functional domains [21]. CBL has also been reported to associate with Golgi membranes together with SRC [22]. We thus investigated the role of CBLC in the maintenance of Golgi organization.

## Results and Discussion

### A targeted screen for Golgi organization identifies CBLC as a major regulator

**Fig 1:**
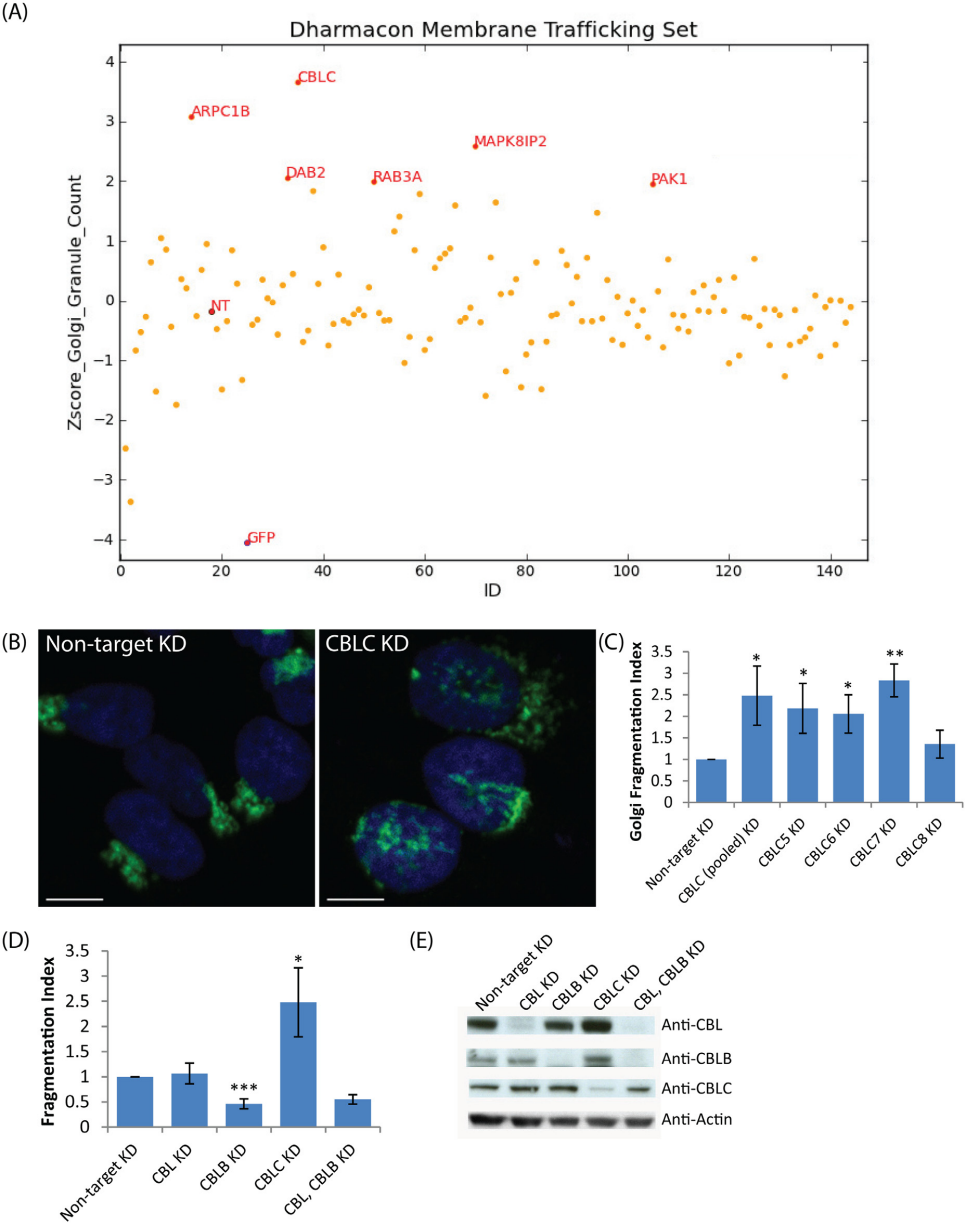
A targeted RNAi screen for Golgi organization identifies CBLC as a Golgi regulator. (A) Plot of granule count of MannII-GFP signal per cell (Z-score) for 120 genes screened. NT: Nontargeting siRNA (B, C) Golgi fragmentation in wild-type HeLa with individual siRNAs. (B) Giantin (green) and Hoechst (blue) staining HeLa cells, CBLC depletion with pool of siRNA. (C) Golgi Fragmentation Index with pool and single siRNAs, measured in triplicates on at least 800 cells per condition. Error bars show SD statistical significance (p) measured by unpaired Student’s t-test. (*) represents p<0.05, (**) represents p<0.01 and (***) represents p<0.001 relative to non-target siRNA transfected cells. Scale bar = 10 μm. (D) Golgi fragmentation Index after targeting Cbl family members, siRNA pools (E) Western blot analysis of CBL, CBLB and CBLC depleted cells.

As a pilot test for perturbation of Golgi apparatus organization, we performed a targeted RNAi screen on 120 genes relevant to membrane trafficking. HeLa cells stably expressing the Golgi-localized GFP-tagged Mannosidase II were reverse transfected with the respective siRNAs, fixed and DAPI stained after 72 hours, then imaged on a high-throughput microscope. Golgi structure was then quantified by counting distinct MannII-GFP fragments (“granules”) using MetaXpress software, and the counts for each gene normalized by Z-score within the plate (Fig 1A).

Among the top ‘fragmenter’ genes, DAB2 is an adaptor protein proposed to inhibit SRC by preventing its phosphorylation at Tyr-416 [23], consistent with data showing that SRC activation leads to Golgi fragmentation [24].

ARPC1B is a component of the Arp2/3 complex that regulates actin polymerization, and actin filaments are known to be important for Golgi morphology and subcellular positioning. The Arp2/3 complex has been shown to be important for Golgi polarity during directed cell migration in 3T3 fibroblasts [25]; WHAMM, the nucleation-promoting factor that regulates Arp2/3, localizes to the Golgi and tubulovesicular transport intermediates and is important for maintaining Golgi structure [26]. PAK1 depletion was also found to cause strong fragmentation in another Golgi-related screen [15].

Altogether, these results suggest that the approach was successful at picking up Golgi regulators. Of these genes, CBLC depletion resulted in the most extensive Golgi fragmentation (Fig 1A).

### CBLC but not CBL or CBLB depletion affects Golgi organization

Next, we depleted CBLC using the four individual siRNAs from the original pool, as well as a different pool of siRNAs and observed extensive fragmentation of Giantin-labelled Golgi (Fig 1B and 1C) for three of the four CBLC siRNAs and the alternative pool, excluding the possibility of an off-target effect.

There are three mammalian Cbl isoforms with highly conserved protein domains: CBL, CBLB, and CBLC. However, neither CBL nor CBLB knockdown induced Golgi fragmentation in HeLa cells (Fig 1D and 1E, S1 Fig) and the Golgi remained compact under these conditions. We also performed double knockdowns of CBL and CBLB and verified efficient protein depletion but still did not observe Golgi fragmentation (Fig 1E).

We next depleted Cbl proteins in another epithelial cell line, Skov-3. Consistent with the observations in HeLa cells, extensive Golgi fragmentation was induced upon knockdown of CBLC and to a lesser extent with CBL, but not with CBLB (S2 Fig). Hence Golgi regulation appears as a new function of CBLC, the least characterized member in the Cbl family.

### CBLC regulates the network organization of the Golgi apparatus

**Fig 2:**
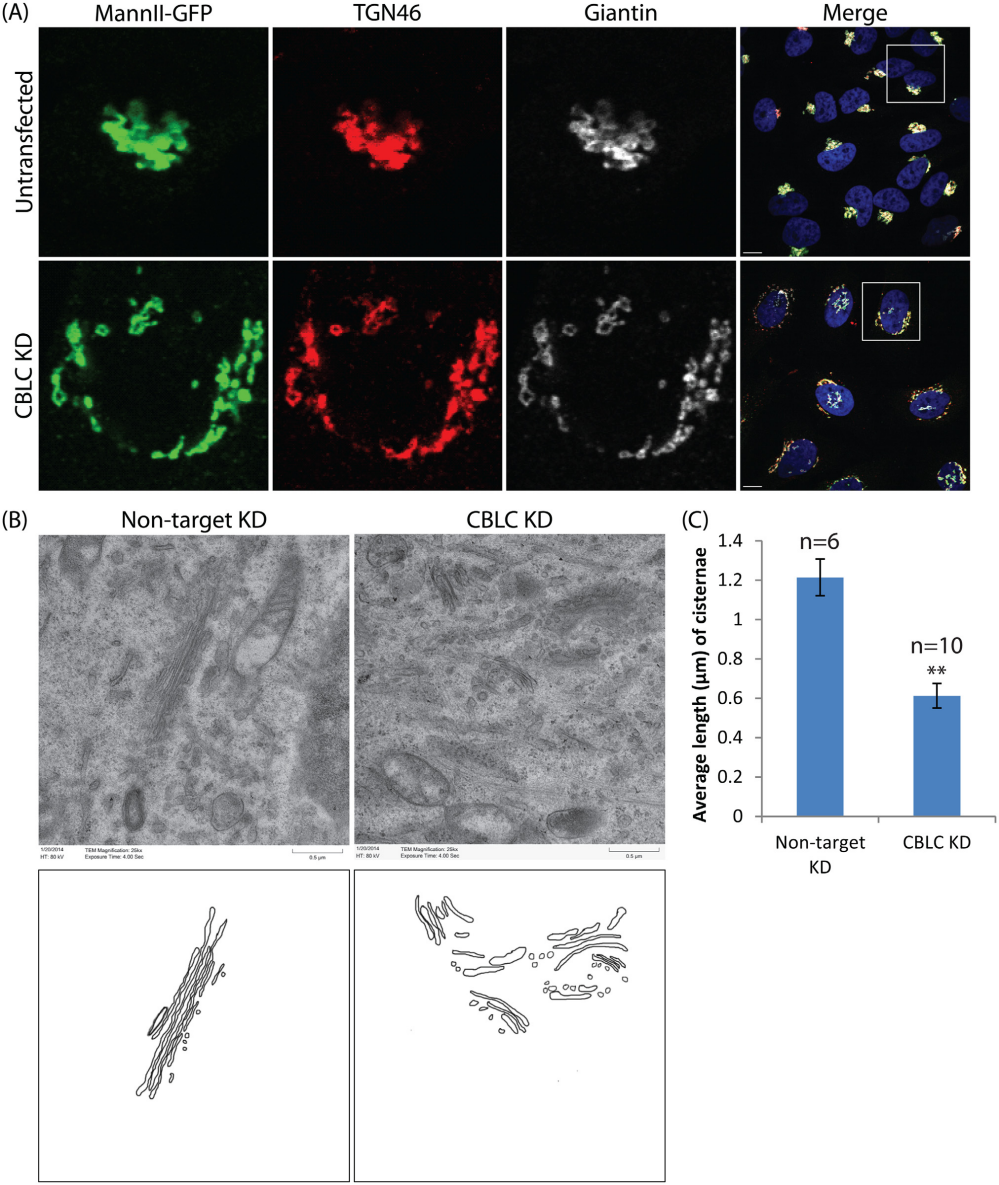
CBLC regulates Golgi ribbon formation. (A) Immunostaining of Golgi using Mannosidase II (green), TGN46 (red) and Giantin (white). Scale bar = 10 μm (B, C) Electron microscopy reveals intact but shorter Golgi stacks in CBLC-depleted cells. Scale bar = 0.5 μm.

To see if CBLC depletion also affects other Golgi cisternae, we knocked down CBLC in HeLa cells stably expressing MannII-GFP which localizes to the medial Golgi, and immunostained the cells for both cis Golgi-localized giantin and trans-Golgi network-localized TGN46. Golgi fragmentation of all compartments was observed, with the fragments colocalizing well with each other (Fig 2A). This indicates that individual Golgi stacks remain intact, and CBLC regulates the network organization of the Golgi rather than specific Golgi cisternae.

To verify this and observe the fragmented Golgi stacks in greater detail, we examined Golgi ultrastructure in control and CBLC-depleted cells using electron microscopy. Intra-Golgi stacking did not appear particularly perturbed, with multiple cisternae remaining closely apposed in control and CBLC-depleted cells (Fig 2B). However, average cisternal length was shorter in the latter (Fig 2C). Thus, CBLC is not required for maintaining intra-stack connections but likely regulates the formation or maintenance of the Golgi ribbon, which requires the lateral linking of cisternae.

The physiological functions of this regulation remain unclear. A preliminary assessment of glycosylation pathways did not reveal any obvious perturbation. Interestingly, CBLC expression is higher in the epithelia of tissues with important secretory functions such as the liver, pancreas, gastrointestinal tract, prostate, and adrenal and salivary glands [27–29], suggesting CBLC might regulate specific aspects of secretion.

### SRC and ARF1 are required for the Golgi fragmentation induced by CBLC depletion

CBLC has been proposed to negatively regulate v-Src, the viral homolog of SRC by ubiquitination [30]. SRC is a membrane-associated tyrosine kinase that localizes at the Golgi when activated [17, 22, 31–34] and has been found in a molecular complex with CBL at the Golgi [22]. SRC activation leads to Golgi fragmentation [24], thus we wondered if Golgi fragmentation caused by CBLC depletion was dependent on SRC.

**Fig 3:**
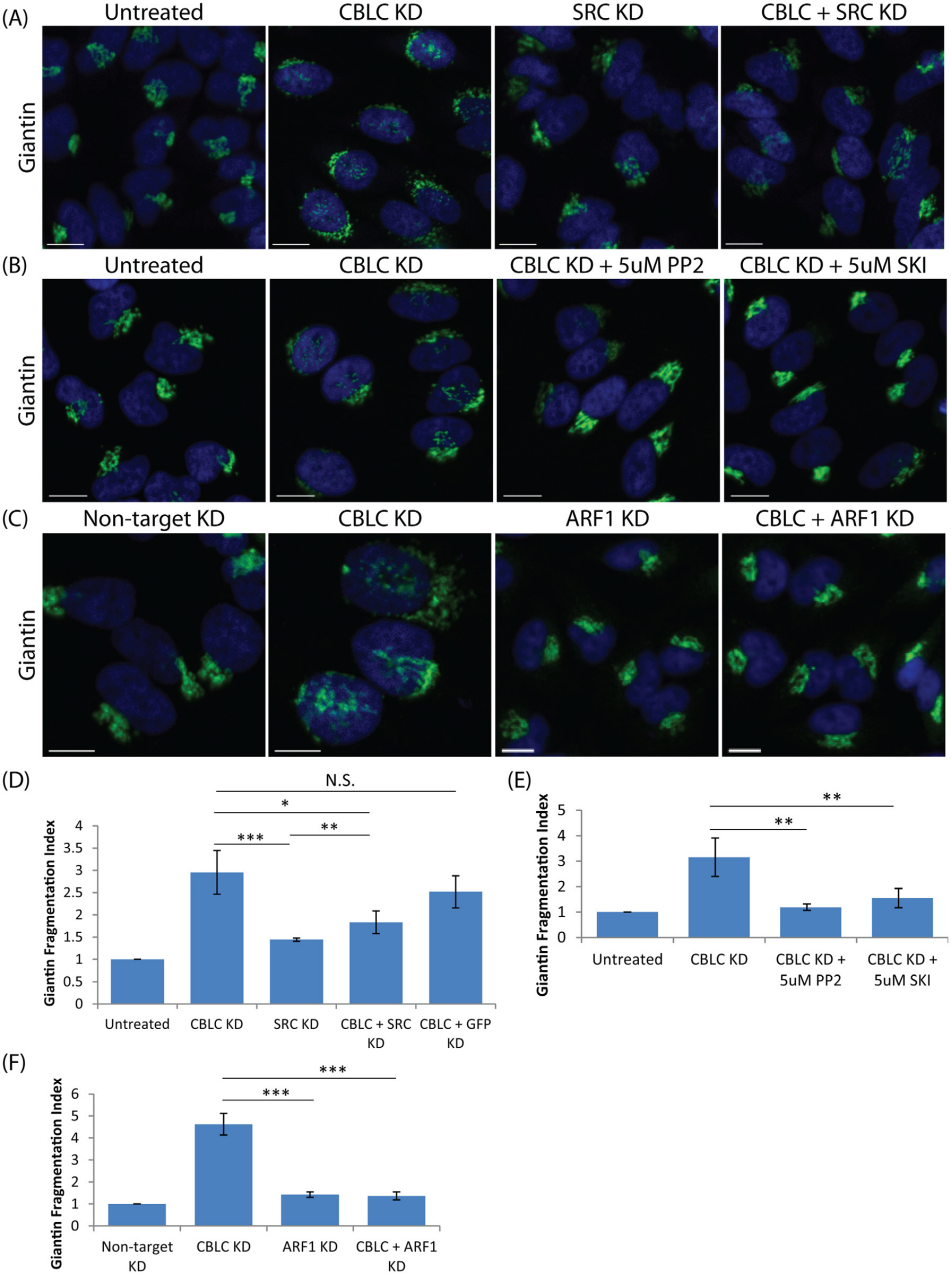
CBLC and SRC antagonistic effect on the Golgi apparatus. Giantin staining in HeLa cells. Fragmentation Index measured in quadruplicates on at least 400 cells per condition. Error bars show SD statistical significance (p) measured by unpaired Student’s t-test. (*) represents p<0.05, (**) represents p<0.01 and (***) represents p<0.001. (A and D) Co-transfection of CBLC and SRC siRNAs in HeLa cells (B and E) Treatment of CBLC-depleted cells with SRC inhibitors PP2 or SKI-1 rescues Golgi morphology. (C and F) Co-transfection of CBLC and ARF1 siRNAs in HeLa cells rescues Golgi morphology. Scale bar = 10 μm.

Cells co-depleted of both CBLC and SRC reverted to a normal Golgi phenotype as seen in control cells (Fig 3A), suggesting that the effect of CBLC depletion is SRC-dependent. Consistently, inhibition of SRC activity for 24 hours after CBLC depletion also resulted in a reversal to normal Golgi morphology (Fig 3B). These data imply that CBLC depletion effect is dependent on SRC activity and suggests that CBLC could act via negative regulation of SRC.

Previous work in our lab has shown that ARF1 is activated downstream of SRC at the Golgi, leading to stimulation of COPI vesicular retrograde trafficking [18]. To test if CBLC’s Golgi regulation is also dependent on this ARF1/COPI pathway, cells were also co-depleted of CBLC and ARF1 and again the Golgi apparatus reverted to a normal morphology (Fig 3C). In sum, CBLC appears to normally antagonize the action of SRC and ARF1.

### CBLC is recruited to the Golgi upon SRC activation

**Fig 4:**
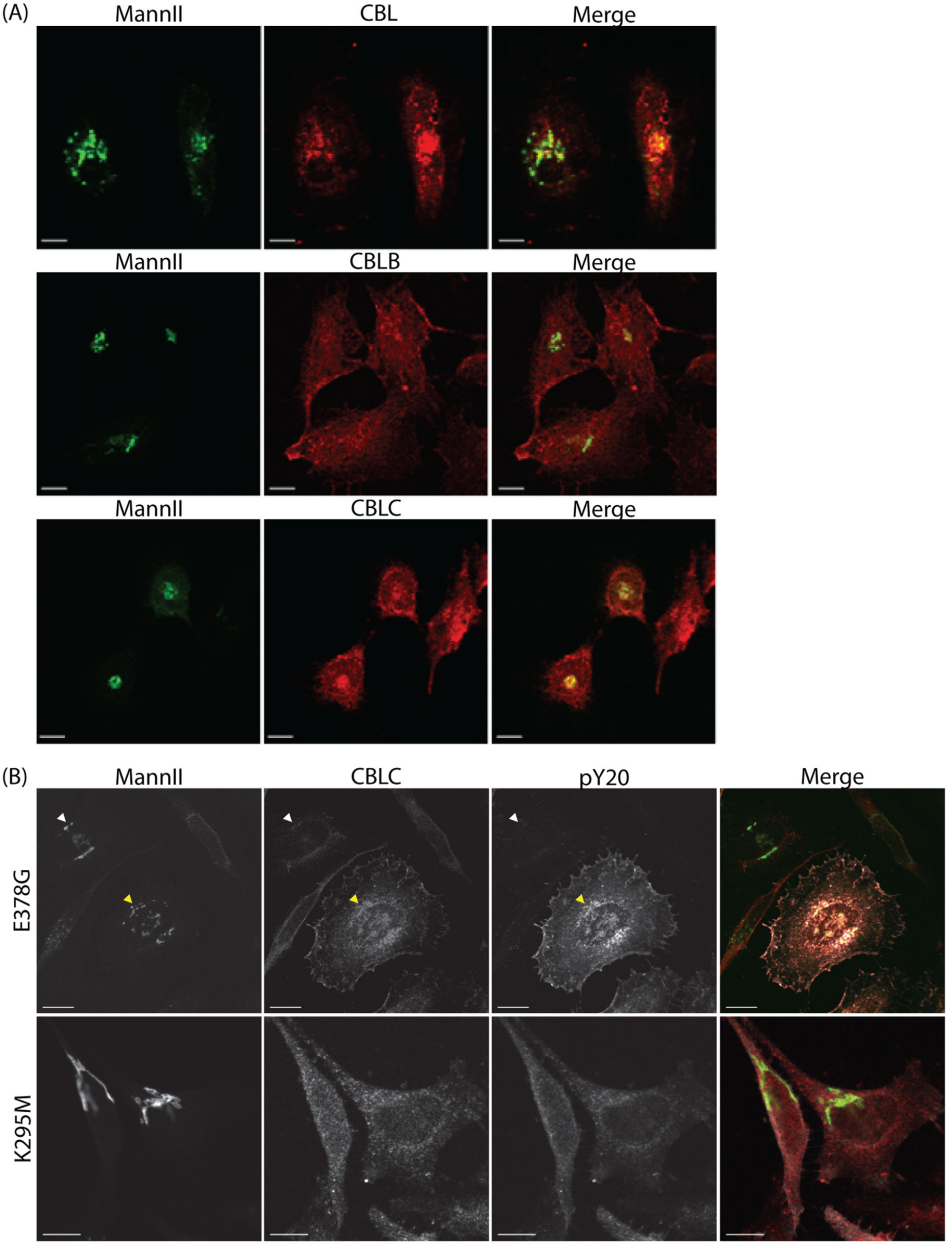
SRC recruits CBLC to the Golgi apparatus. (A) CBL and CBLC localize to the Golgi apparatus. MannII-GFP and endogenous CBL, CBLB, or CBLC staining in HeLa cells. (B) SRC activation enhances recruitment of CBLC to the Golgi apparatus. Cells were transfected with either constitutively active SRC E378G or inactive SRC K295M. Staining for tyrosine-phosphorylated proteins stained with pY20 (white) to report SRC transfection and for endogenous CBLC (red). CBLC level at the Golgi was increased in cells overexpressing active SRC (yellow arrow) but not inactive SRC. Scale bar = 10 μm.

We next tested if CBLC protein is present at the Golgi apparatus. Immunofluorescence staining of endogenous Cbl proteins showed that while all three Cbl proteins are detected in the cytoplasm, only CBL and CBLC, but not CBLB, localize to the Golgi apparatus (Fig 4A).

In addition, we looked at the localization of endogenous CBLC in HeLa cells transfected with either constitutively active (E378G) or kinase-dead (K295M) SRC mutants. Cells overexpressing active SRC but not inactive SRC showed increased CBLC levels at the Golgi (Fig 4B), suggesting that SRC activation enhances recruitment of CBLC to the Golgi apparatus.

The interaction of Cbl family proteins with SRC [22, 30, 35–39] is well-established. CBL’s regulation of SRC is complex, with CBL able to bind SRC through both its PTB domain and proline-rich region, and regulating SRC not only negatively, but positively as well [40].

The involvement of ARF1 in the pathway negatively regulated by CBLC suggests a role of CBLC in modulating COPI trafficking. Interestingly, COPI tubules have been implicated in the maintenance of Golgi ribbon [41]. Furthermore, ARF1 has been shown to be required for mitotic Golgi disassembly [42], which is congruent with its role in Golgi fragmentation induced by CBLC depletion.

### CBLC regulation of the Golgi network is dependent on its RING domain but not on SRC degradation

**Fig 5:**
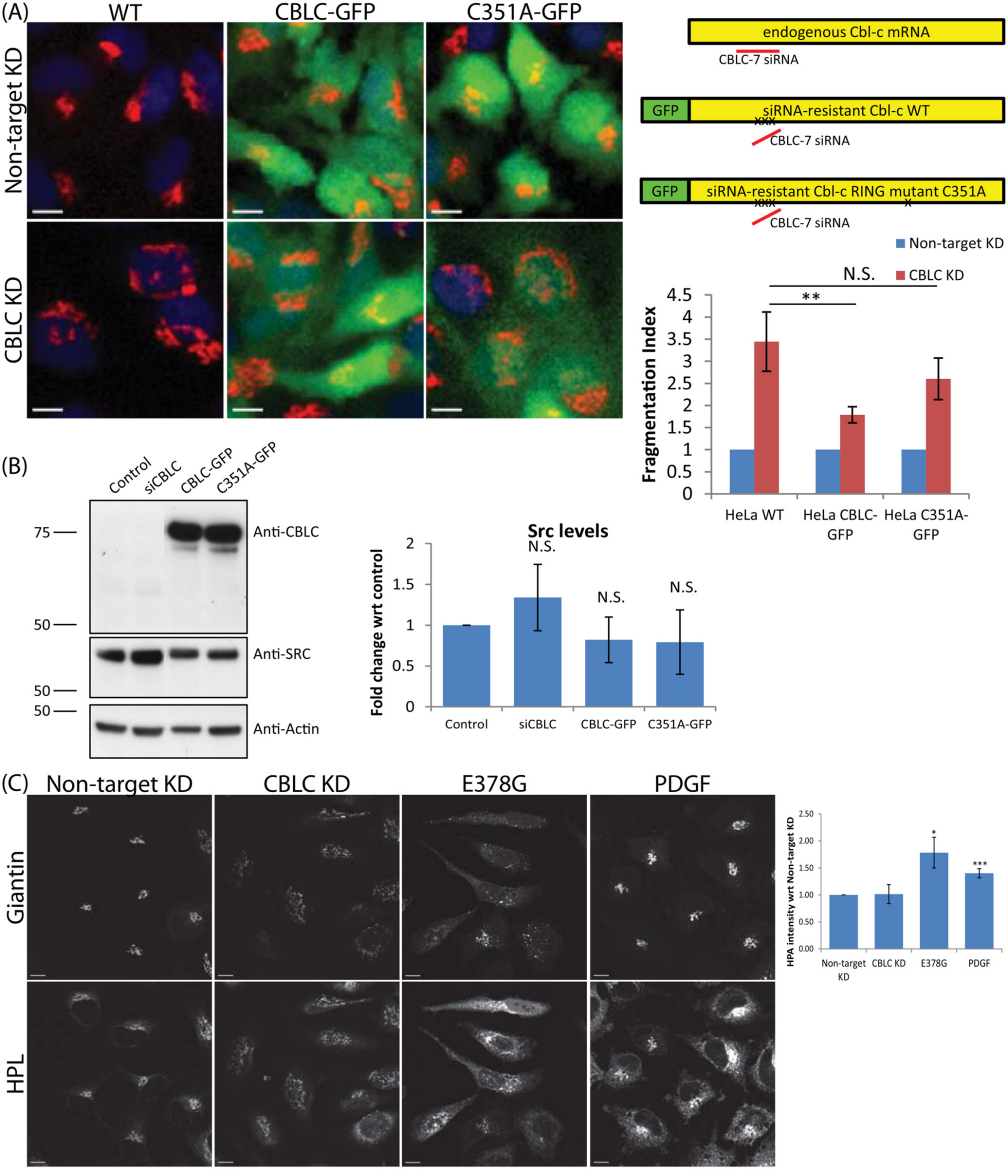
CBLC ubiquitin ligase activity is required for Golgi but not SRC levels regulation. (A) Requirement of CBLC RING domain. HeLa wild type (WT) or stably expressing siRNA-resistant-CBLC (CBLC-GFP) or siRNA-resistant-CBLC RING mutant (C351A-GFP) cells were transfected with deconvoluted CBLC-7 siRNA. Golgi stained with Giantin. Top right: Schematic diagram, exogenous CBLC contains three-point mutations at CBLC-7 siRNA-binding site. Bottom right: Fragmentation Index measured in quadruplicates on at least 400 cells per condition. Error bars show SD statistical significance (p) measured by unpaired Student’s t-test. (**) represents p<0.01 relative to non-target siRNA transfected cells. Scale bar = 10μm. (B) SRC levels analysis by western blot upon CBLC silencing or overexpressing. (C) CBLC depletion does not activate GALNTs relocation. pY20 immunostaining marks SRC-activated cells, Golgi labelled with Giantin, and GALNTs activity revealed by HPL staining. HeLa cells transfected with non-target siRNA, CBLC siRNA, or constitutively active SRC E378G plasmid, or stimulated with PDGF. Scale bar = 10 μm.

CBLC is an E3 ubiquitin ligase with a RING domain, which is critical for ubiquitination. To test if ubiquitin ligase activity is required for the regulation of the Golgi, we generated HeLa cells stably expressing exogenous wild type or RING mutant (C351A) CBLC, with silent point mutations rendering them resistant to CBLC-7 siRNA knockdown (Fig 5A). Next, we depleted endogenous CBLC in these cells with CBLC-7 siRNA. As expected, we found that expression of exogenous wild type CBLC protected the Golgi from fragmentation. By contrast, the Golgi fragmented in cells expressing C351A CBLC (Fig 5A), indicating that ubiquitin ligase activity is required for maintenance of the Golgi network.

Our data show that SRC activation enhances recruitment of CBLC to the Golgi apparatus, and that CBLC has antagonizing effects to SRC on Golgi organization. Furthermore, it has been shown that CBLC reduces v-Src levels by ubiquitin-dependent degradation [30].

Therefore, CBLC might negatively regulate SRC by inducing its ubiquitination and degradation. We quantified SRC levels in HeLa cells depleted of or overexpressing CBLC. We found no significant effect on SRC levels upon CBLC depletion, in both immunoblotting (Fig 5B) and immunofluorescence assays (S3 Fig). Upon CBLC overexpression, there is also no significant decrease in SRC levels as compared to untransfected cells (Fig 5B). No noticeable difference in SRC levels was detected upon overexpressing wild type or RING mutant CBLC.

Overall, these results suggest that CBLC does not regulate total SRC levels in HeLa cells. It is possible that CBLC specifically ubiquitinates a Golgi-localized fraction of SRC that is too small for us to detect.

Cullin-5, another E3 ubiquitin ligase, has also been shown to affect levels of active but not inactive SRC [43]. In fact, these authors proposed that Cullin-5 rather than Cbl proteins is a negative regulator of SRC at the general cellular level. Like Cbl proteins, Cullin-5 is an E3 ubiquitin ligase with a RING domain. Based on these similarities, we investigated whether Cullin-5 regulates Golgi organization, however, Cullin-5-depleted cells displayed an intact-looking Golgi (S4 Fig).

Thus, of the four proposed negative regulators of SRC tested in our study, only CBLC regulates Golgi organization, apparently not by directly inducing SRC degradation. Consistent with our data, *in vitro* E3 ligase assays using recombinant GST-CBLC constructs and purified active SRC found that the addition of CBLC did not alter SRC levels [38]. An alternative hypothesis is thus that CBLC action might not require protein degradation or rather affect proteins downstream of SRC.

### CBLC counteracts SRC effect at the Golgi

SRC activation at the Golgi triggers at least two distinct events: fragmentation and the relocation of GALNTs from the Golgi to the ER [8, 18, 24]. This relocation can be measured by the increased staining intensity of HPL, a lectin that binds to the sugar residue added by GALNTs.

To elucidate the relationship between CBLC and SRC activities at the Golgi, we compared Golgi morphology and HPL staining upon CBLC knockdown and SRC activation, using either PDGF stimulation to induce a moderate SRC activation or expression of SRCE378G mutant to mimic high levels of activation. Consistent with published data, HPL intensity increased and its localization spread from just the Golgi to both Golgi and ER upon SRC activation (Fig 5C). In addition, the Golgi fragmented upon high SRC activation (Fig 5C).

In contrast, in CBLC-depleted cells, the Golgi fragmented but there was no significant change in HPL intensity or localization. Thus, while CBLC knockdown causes extensive fragmentation as high SRC activation does, the phenotypes differ at the level of GALNTs relocation.

Taken together, these results indicate that while CBLC’s maintenance of the Golgi network antagonizes SRC activity, it likely does not directly downregulate SRC, even at the Golgi. One possibility is that CBLC ubiquitinates a substrate downstream of SRC and important for Golgi ribbon structure.

Apart from the canonical role of ubiquitination in protein degradation, it is increasingly well-established that ubiquitination also plays critical roles in membrane trafficking [44, 45], commonly by serving as a signal to recruit proteins containing ubiquitin-binding domains. For example, monoubiquitination of COPII coat component Sec31 was shown to form large COPII coats, critical for efficient ER-Golgi trafficking of collagen [46].

Interestingly, de-ubiquitination of beta’-COP was found to be important for Golgi-ER retrograde traffic [47]. Significantly, COPI-dependent tubules, formed in opposing balance to vesicles, have recently been found to play a major role in Golgi ribbon formation [41]. This raises the intriguing possibility that ubiquitination of COPI may play a role in CBLC regulation of the Golgi ribbon. We tend to observe more vesicles surrounding the Golgi in our ultrastructural images of CBLC-depleted cells, possibly reflecting a switch from tubule to vesicle formation. An additional suggestion of the potential role of COPI is the report that a major pool of the adaptor CIN85 (Cbl-interacting protein of 85 kDa) is associated with the Golgi, mainly with a subpopulation of COPI-coated vesicles [48]. Clearly, more work is required to decipher how CBLC is able to regulate Golgi ribbon structure.

## Materials and Methods

### Antibodies

Anti-actin monoclonal, Anti-Giantin polyclonal, and Anti-phosphotyrosine (pY20) monoclonal were purchased from Abcam. Anti-SRC polyclonal and Anti-TGN46 polyclonal were purchased from Santa Cruz and Serotec respectively. Anti-CBL polyclonal and Anti-CBLB monoclonal were a gift from G. Guy (Institute of Molecular and Cell Biology, Singapore), Anti-CBLC polyclonal was a gift from M. Kim (Institute of Medical Science, Tokyo, Japan) while another Anti-CBLC polyclonal was purchased from Abnova. Helix pomatia Lectin A (HPL) conjugated with 647 nm fluorophore was purchased from Invitrogen.

### Cloning

Plasmids encoding full-length untagged SRC with point mutant E378G, N-terminus mCherry-tagged SRC E378G, and N-terminus mCherry-tagged SRC with point mutant K295M were a gift from S. Wang (Institute of Molecular and Cell Biology, Singapore).

Total RNA was extracted from HeLa cells and full-length CBLC (GenBank/EMBL/DDBJ accession no. NM_012116) was synthesized using SuperScript-III (Invitrogen). The cDNA was PCR amplified using attB-containing primers and cloned into an attP-containing donor vector (pDONR221) using BP-clonase II (Invitrogen). C351A point mutant was introduced into CBLC-pDONR221 entry clone by PCR using overlapping mutagenic primers and KOD polymerase (Merck). CBLC wild type and CBLC C351A were subcloned into pLenti6.3_NemGFP_DEST using LR-clonase II (Invitrogen). All constructs were verified by sequencing before use.

### Cell culture

HeLa and HeLa-Mannosidase II-GFP human cell lines were a gift from V. Malhotra (Centre for Genomic Regulation, Barcelona, Spain). Skov3 (human ovarian epithelial cells, ATCC^®^ HTB 77^TM^) was a gift from E. Bard (IMCB, Singapore).

All cells were grown at 37°C in a 10% CO_2_ humidified incubator. HeLa and Skov-3 cells were grown in high glucose DMEM supplemented with 10% FBS.

### Lentivirus production and generation of CBLC-GFP stable cell lines

CBLC (wild-type)- and C351A-GFP were stably infected into HeLa cells by lentiviral infection as described in Gill et al 2013.

### siRNA screening

#### siRNA transfection

The screen library was the Dharmacon “Membrane Trafficking” library; siGENOME SMARTpool. Reverse siRNA transfection was performed by pre-mixing HiPerFect® Transfection reagent (Qiagen) with OptiMEM (Invitrogen) for 5 minutes, then adding the mixture to the siRNA for complexation for 20 minutes, and lastly adding 2000 HeLa MannII-GFP cells per well with the Multidrop combi (Thermo-Fisher). siRNA transfections were performed in duplicate wells. Cells were incubated at 37°C in a 10% CO_2_ humidified incubator for 72 hours prior to harvesting for analysis.

#### Immunofluorescence and imaging

Cells were fixed with 4% paraformaldehyde in D-PBS for 10 minutes, washed with D-PBS and permeabilized with 0.2% Triton X-100 for a further 10 minutes. Cells were then stained with Hoechst 33342 (Invitrogen) diluted in 2% FBS in D-PBS for 20 minutes, then washed three times for 5 minutes with D-PBS before high-throughput confocal imaging using ImageXpress Ultra (MDS Analytical Technologies).

#### Image analysis

Images were analyzed using MetaXpress software (Molecular Devices). Transfluor HT module was used to quantify Mannosidase II-GFP granule count and cell number. The Z-score for granule count per cell was calculated as a measure of Golgi fragmentation, given by z = (x- μ)/σ, where x is the granule count of the individual sample, and μ and σ the mean and standard deviation of granule counts of all samples, respectively.

### RNA interference

Dharmacon ON-TARGETplus SMARTpool siRNAs (Dharmacon, now Thermo Fisher) were used for silencing CBL, CBLB, CBLC, SRC, ARF1 and CUL5 in HeLa and Skov-3 cell lines. Allstars non-target siRNA (Qiagen) was used as a negative control. Reverse siRNA transfection was performed by incubating HiPerFect® Transfection reagent with OptiMEM for 5 minutes at room temperature before the addition of 25nM siRNA. The transfection complex was incubated for 20 minutes on shaker at 200 rpm, and added onto the wells. Trypsinized cells diluted in DMEM supplemented with 10% FBS were added to the wells containing the transfection complex and incubated at 37°C in a 10% CO_2_ humidified incubator for 72 hours prior to harvesting for analysis.

### Transient expression of plasmid DNA in mammalian cells

Plasmids were transfected into HeLa cells using FuGENE® HD (Promega) transfection reagent according to manufacturer’s instructions. Cells were incubated at 37°C in a 10% CO_2_ humidified incubator for 16 hours before harvesting for further analysis.

### Cell lysis and Immunoprecipitation

Cells were lysed using lysis buffer (containing 0.5% NP-40 Alternative (Calbiochem), 200 mM NaCl and 50 mM Tris buffer, pH 8.0) in the presence of protease (Roche) and phosphatase (Roche) inhibitors at 4°C on a 20 rpm shaker for 30 minutes. Lysates were clarified by centrifugation at 14,000g for 10 minutes at 4°C. Lysates were incubated with appropriate antibodies for 2 hours, followed by addition of pre-cleared agarose beads and lysates were incubated for a further 2 hours. Agarose beads were washed three times with wash buffer (containing 0.5% NP-40 Alternative (Calbiochem), 100 mM NaCl and 50 mM Tris buffer, pH 8.0) and immunoprecipitated protein was resuspended in 2X SDS sample buffer and heated at 95°C for 5 minutes. Samples were loaded onto 4–12% NuPAGE® Novex Bis-Tris Gel (Invitrogen) and gel electrophoresis was performed before analysing using Western blot.

### Immunoblotting

PVDF membranes were blocked in blocking buffer [TBS with 3% BSA and 0.1% Tween 20] at room temperature for 30 minutes, and incubated on shaker at 20 rpm for 1–2 hours at room temperature (or at 4°C overnight) with appropriate primary antibodies diluted in blocking buffer. After 3 washes with TBS-T [TBS with 0.1% Tween 20] with each wash being 10 minutes, membranes were incubated on shaker at 20 rpm with appropriate IgG conjugated with horseradish peroxidase (GE Healthcare) diluted in blocking buffer for 1 hour at room temperature. After three washes with TBS-T as above, membranes were subjected to chemiluminescence detection with an ECL kit (GE Healthcare). To re-probe the same membrane with another antibody, the membrane was incubated in stripping buffer [62.5mM Tris-HCl pH 6.7, 100mM ß mercaptoethanol, 2% SDS] at 55°C for 20 min, washed thrice with TBS-T, followed by blocking.

### Immunofluorescence

Cells were fixed with 4% paraformaldehyde in D-PBS for 10 minutes, washed with D-PBS and permeabilized with 0.2% Triton X-100 for a further 10 minutes. Cells were incubated with appropriate antibodies diluted in 2% FBS in D-PBS for a duration ranging from 1 hour at room temperature to overnight at 4°C, depending on the strength of the antibodies. Cells were washed thrice for 10 minutes using 2% FBS in D-PBS and subsequently stained with secondary Alexa Fluor-conjugated antibodies (Invitrogen) and Hoechst 33342 for 15 minutes. Cells were washed thrice for 10 minutes using D-PBS. Cells that were seeded onto coverslips were mounted onto glass slides using FluorSave (Merck) and imaged at room temperature using an inverted FluoView confocal microscope (model IX81; Olympus) coupled with a CCD camera (model FV11) with a 60x objective (U Plan Super Apochromatic [UPLSAPO]; NA 135) under Immersol oil. Images were acquired and processed using Olympus FV10-ASW software. Cells that were seeded onto 96-well plates were imaged at room temperature using ImageXpress Ultra (IXU) confocal microscope (Molecular Devices) with a 10x or 20x objective (Nikon). Images were acquired and processed using MetaXpress software (Molecular Devices).

The immunofluorescence protocol was slightly modified for immunostaining of Cbl proteins. Cells were fixed as described above. Cells were permeabilized 0.02% Triton X-100 in PBS for 1 hour, antibodies were diluted in 2% FBS, 0.02% Triton X-100 in D-PBS for subsequent incubations, and cells were washed using 0.02% Triton X-100 in D-PBS.

### Golgi fragmentation quantification

HeLa cells were seeded onto a 96-well clear and flat-bottomed black imaging plate (BD). siRNA reverse transfection, cell fixation, staining and imaging were performed as described above. Images were processed using Granularity module of MetaXpress analysis software (MDS Analytical Technologies) and “granules per cell” information was extracted. Golgi compartment was delineated using either of these medial-Golgi markers, Mannosidase II or Giantin, and Golgi staining was acquired using IXU confocal microscope. Number of granules per cell was quantified using Granularity module of MetaXpress analysis software (MDS Analytical Technologies). Approximately 800 cells were quantified per condition. Cells with no granule count (unstained cells) or more than 100 granule count (background noise) were excluded. Relative frequency distribution of the number of granules per cell for each condition was tabulated. “Fragmentation Index” (FI) formula was developed where ${\text{FI}}_{\text{sample}}=\frac{\sum \left[{\left(\text{granule count}\right)}^{2}\text{ x relative frequency}\right]}{FI\left(control\right)}$ to improve the representation of extent of Golgi fragmentation.

### PDGF stimulation in HeLa cells

HeLa cells were seeded onto 96-well clear and flat-bottomed black imaging plate (BD) or glass coverslips in a 24-well plate (Nunc) and incubated overnight at 37°C in a 10% CO_2_ humidified incubator to allow adherence of cells to the bottom of wells or coverslips. Medium was then aspirated and replaced with serum-free DMEM. After serum starvation for 16 hours, medium was aspirated and replaced with serum-free DMEM containing 50 ng/ml PDGF (Invitrogen) and incubated for 2 hours at 37°C in a 10% CO_2_ humidified incubator before harvesting.

### SRC inhibition in CBLC-depleted cells

Reverse transfection of siRNA was performed as described above in HeLa cells seeded onto a 96-well clear and flat-bottomed black imaging plate (BD). After 48 hours of siRNA transfection, medium was aspirated, replaced with fresh medium containing 5 μM SRC inhibitors PP2 or SRC inhibitor kinase 1 SKI-1 (Sigma) and incubated at 37°C in a 10% CO_2_ humidified incubator for a further 24 hours before fixation.

## Supporting Information

S1 Fig CBLC but not CBL or CBLB knockdown induces Golgi fragmentation. Giantin (green) and nuclei (blue) staining in siRNA-transfected HeLa cells.
(PDF)

S2 Fig CBLC knockdown effect is conserved across different cell lines. (A) Giantin (green) and nuclei (blue) staining in siRNA-transfected Skov-3 cells. CBLC siRNA pool and individual siRNAs (CBLC5, CBLC6, CBLC7). (B,C) Quantification of fragmentation in Skov-3 cells. (D) Western blot analysis Cbl proteins in HeLa and Skov-3 cell lines. Scale bar = 10 μm.
(PDF)

S3 Fig SRC levels are not significantly affected upon CBLC depletion. Giantin (green) and SRC (red) staining in siRNA-transfected HeLa cells. Quantification of SRC intensity on at least 400 cells. Scale bar = 10 μm.
(PDF)

S4 Fig Depletion of Cullin-5 does not induce Golgi fragmentation. Top: Giantin (green) staining in siRNA-transfected HeLa cells. Scale bar = 10μm. Bottom left: Fragmentation Index measured in triplicates on at least 400 cells per condition. Error bars show SD statistical significance (p) measured by unpaired Student’s t-test. (***) represents p<0.001 relative to non-target siRNA transfected cells. Bottom right: Western blot analysis of CBLC and CUL5 knock-downs. Two bands were detected upon blotting with CBLC antibody, with a specific upper band (black arrow) and a non-specific lower band (white arrow).
(PDF)
