# Supplementary figures and images for: The Ubiquitin Ligase CBLC Maintains the Network Organization of the Golgi Apparatus
Source: PLoS One. 2015 Sep 22;10(9):e0138789. doi: 10.1371/journal.pone.0138789 (PMC4579092; doi:10.1371/journal.pone.0138789)

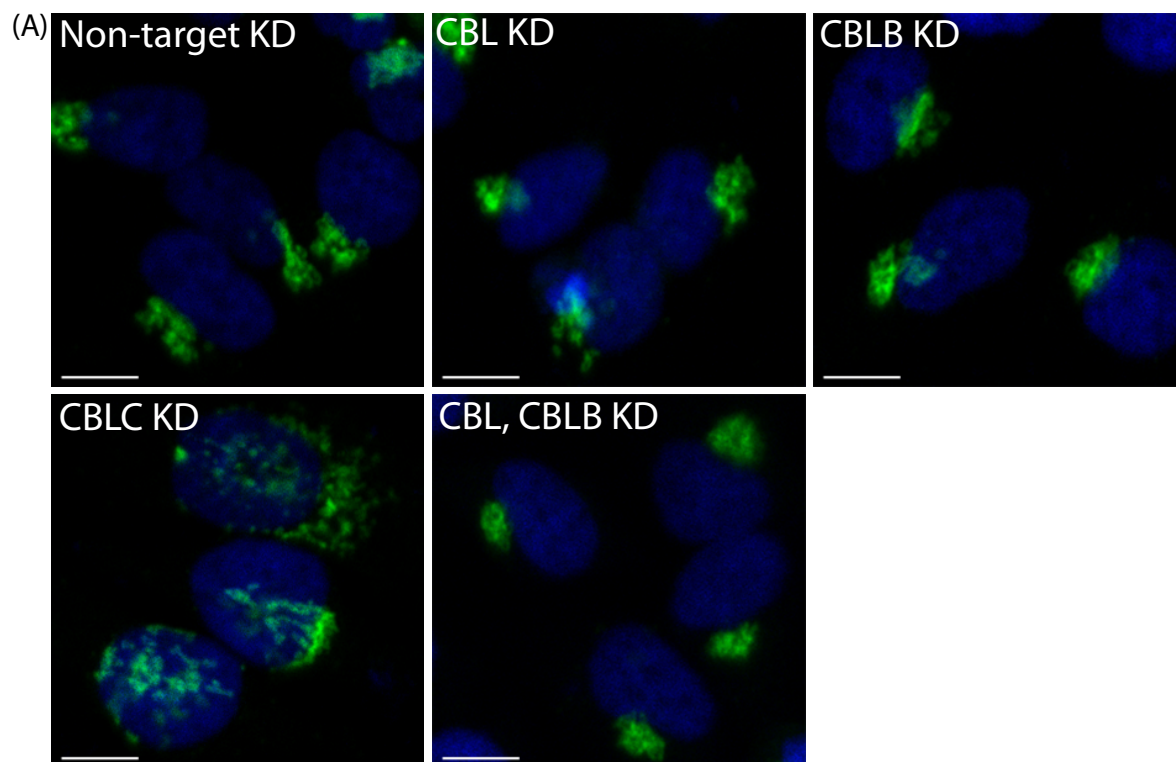

Supplementary Figure 1

Supplement: S1 Fig — Giantin (green) and nuclei (blue) staining in siRNA-transfected HeLa cells. (PDF) [file pone.0138789.s001.pdf]

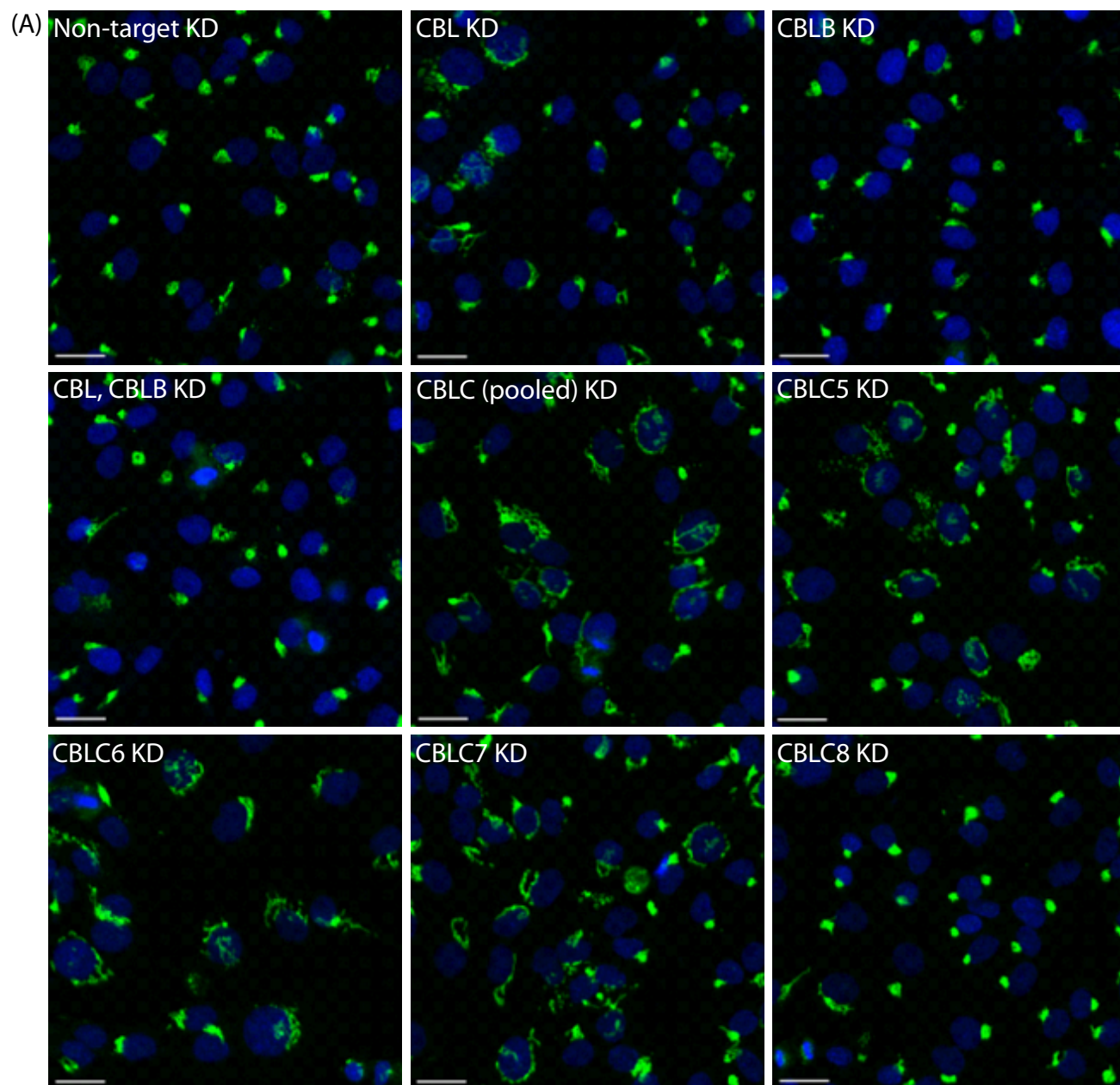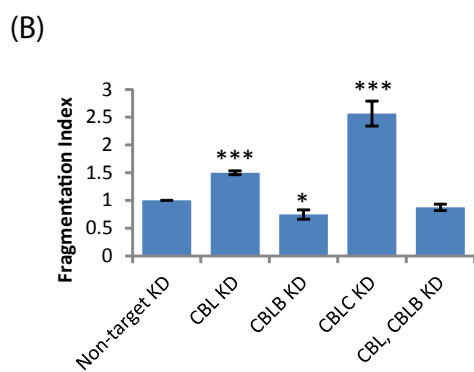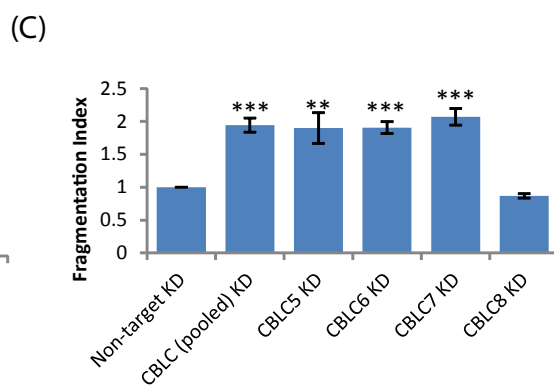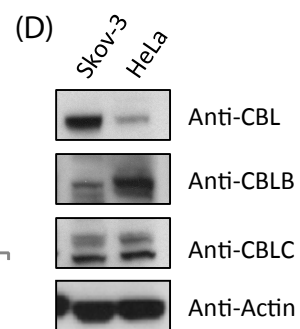

Supplementary Figure 2

Supplement: S2 Fig — (A) Giantin (green) and nuclei (blue) staining in siRNA-transfected Skov-3 cells. CBLC siRNA pool and individual siRNAs (CBLC5, CBLC6, CBLC7). (B,C) Quantification of fragmentation in Skov-3 cells. (D) Western blot analysis Cbl proteins in HeLa and Skov-3 cell lines. Scale bar = 10 μm. (PDF) [file pone.0138789.s002.pdf]

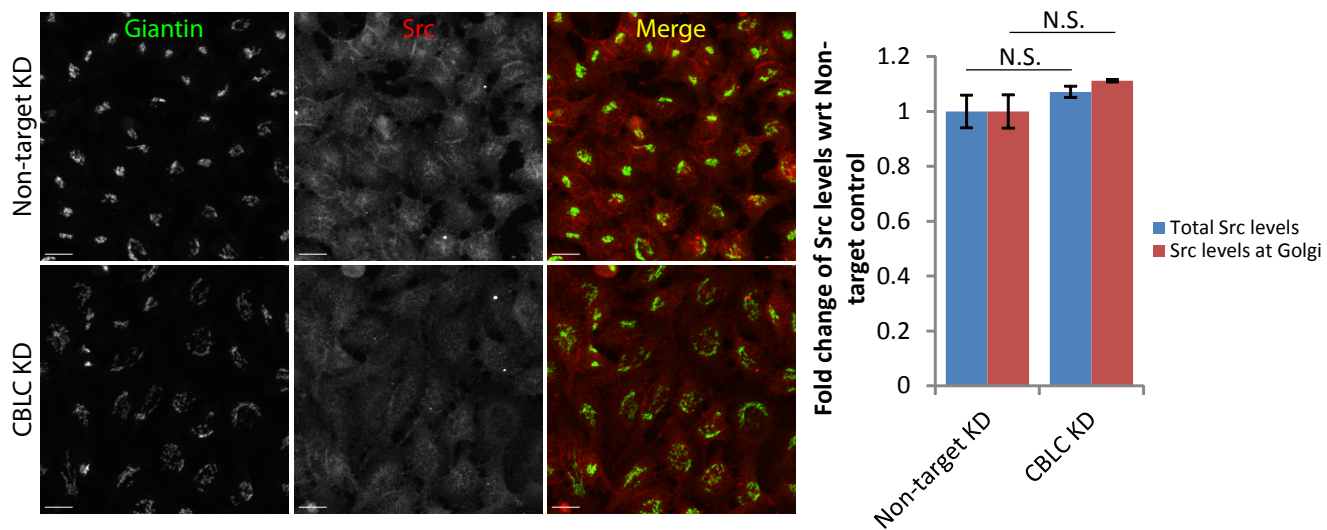

Supplementary Figure 3

Supplement: S3 Fig — Giantin (green) and SRC (red) staining in siRNA-transfected HeLa cells. Quantification of SRC intensity on at least 400 cells. Scale bar = 10 μm. (PDF) [file pone.0138789.s003.pdf]

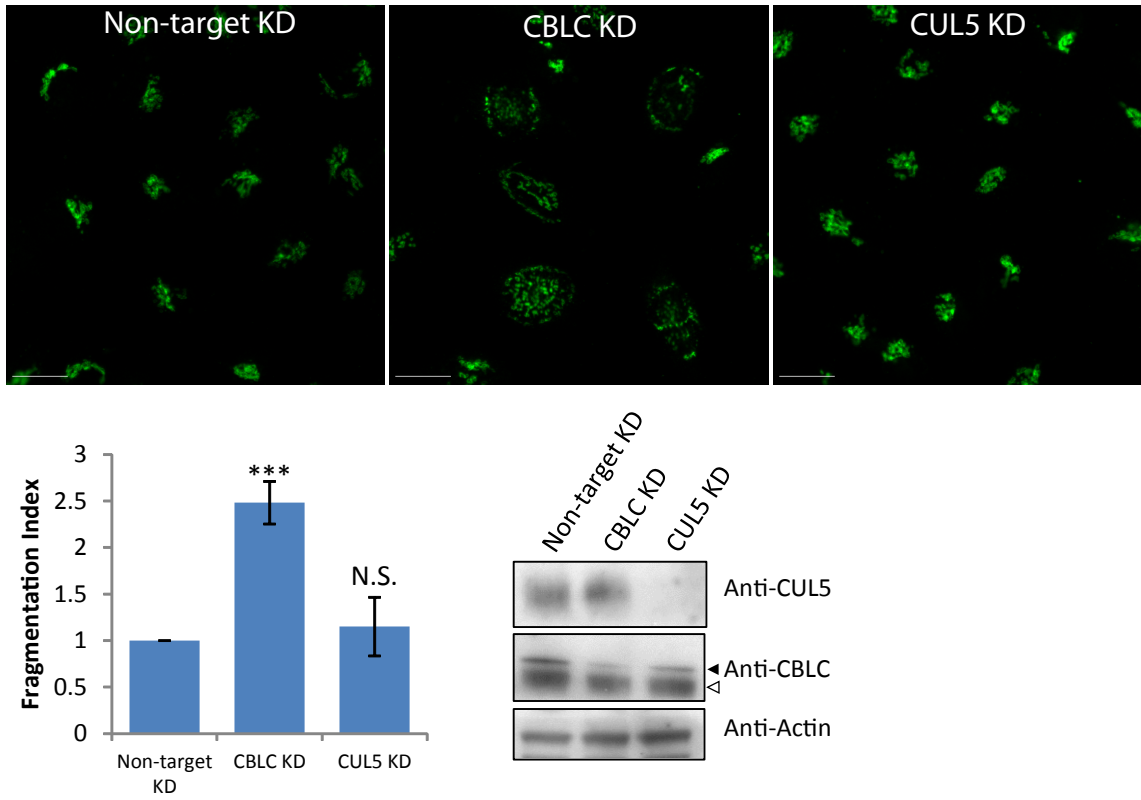

Supplementary Figure 4

Supplement: S4 Fig — Top: Giantin (green) staining in siRNA-transfected HeLa cells. Scale bar = 10μm. Bottom left: Fragmentation Index measured in triplicates on at least 400 cells per condition. Error bars show SD statistical significance (p) measured by unpaired Student’s t-test. (***) represents p<0.001 relative to non-target siRNA transfected cells. Bottom right: Western blot analysis of CBLC and CUL5 knock-downs. Two bands were detected upon blotting with CBLC antibody, with a specific upper band (black arrow) and a non-specific lower band (white arrow). (PDF) [file pone.0138789.s004.pdf]
